# Women Trust Their OBGYNs to Provide Preexposure Prophylaxis: An Opportunity for HIV Prevention

**DOI:** 10.3389/frph.2022.832287

**Published:** 2022-03-15

**Authors:** Antoinette A. Danvers, Emma Chew Murphy, Karina Avila, Tatiana Gonzalez-Argoti, Angelic Rivera Edwards, Susie Hoffman, Joanne E. Mantell, Laurie J. Bauman, Siobhan M. Dolan

**Affiliations:** ^1^Department of Obstetrics and Gynecology and Women's Health, Montefiore Medical Center, Albert Einstein College of Medicine, Bronx, NY, United States; ^2^Departments of Pediatrics and Psychiatry and Behavioral Health, Albert Einstein College of Medicine, Bronx, NY, United States; ^3^Montefiore School Health Program, Department of Pediatrics, Montefiore Medical Center, Albert Einstein College of Medicine, Bronx, NY, United States; ^4^Department of Epidemiology, Joseph L. Mailman School of Public Health at Columbia University, New York, NY, United States; ^5^Department of Psychiatry, HIV Center for Clinical and Behavioral Studies, New York State Psychiatric Institute, Columbia University Irving Medical Center, New York, NY, United States; ^6^Department of Obstetrics, Gynecology and Reproductive Science, Icahn School of Medicine at Mount Sinai and Mount Sinai Health System, New York, NY, United States

**Keywords:** preexposure prophylaxis, reproductive health, gynecology, OBGYN and PrEP, HIV prevention, AIDS

## Abstract

**Objective:**

The objective of this study was to understand how women perceive the role of their Obstetrician and Gynecologist (OBGYN) in screening for and providing preexposure prophylaxis (PrEP) for HIV prevention.

**Methods:**

We recruited women ages 18–45 years receiving obstetric or gynecological care at an academic medical center in the Bronx, NY. Thirty participants were enrolled: 10 seeking care for family planning, 10 seeking prenatal care, and 10 seeking care for a sexually transmitted infection. We screened participants for HIV acquisition risk using a PrEP screening tool. We conducted face-to-face, semi-structured interviews, which were audio-recorded, transcribed, and entered into Dedoose for analysis of themes using a grounded theory approach.

**Results:**

Sixty percent of the participants were Latinx and 33% African American. Seventy percent had one or more risk factors for HIV acquisition based on the PrEP screening tool, indicating they would benefit from a PrEP discussion. Three main themes emerged from the analysis of interview data. Participants viewed OBGYNs as experts in sexual and reproductive healthcare and believed they were experts in PrEP. Participants were concerned about “PrEP stigma”, being judged by their clinicians as being sexually promiscuous if they expressed a need for PrEP. Lastly, when participants trusted their OBGYN, that trust became a facilitator for women to consider PrEP and offset stigma as a barrier to identifying patients who are candidates for PrEP.

**Conclusion:**

Women established in care with an OBGYN are enthusiastic about having access to PrEP services incorporated into their sexual and reproductive healthcare. A universal approach to HIV prevention would avert stigma surrounding HIV care and prevention.

## Introduction

According to the United States (U.S.) Centers for Disease Control and Prevention's 2018 HIV Surveillance Report, about 20% of new HIV infections in the U.S. occur in women. The report reveals significant racial disparities in the incidence of new HIV infections, with African American women accounting for almost 60% of new HIV infections among women. This means that African American women have a 1 in 54 lifetime risk of receiving an HIV diagnosis (1). In addition, the lifetime risk for Hispanic women is 1 in 256 compared to a 1 in 941 lifetime risk for white women (1). The persistently high rates of HIV among African Americans and Latinxs are a major concern.

Preexposure prophylaxis (tenofovir plus emtricitabine, PrEP) significantly decreases the risk of HIV transmission (2), making it a critical opportunity for women at risk. The safety and effectiveness of PrEP are well-established (3). Clinical trials show that PrEP is not associated with pregnancy-related complication (2) and does not interact with hormonal contraception (4). Thus, increasing the uptake of PrEP among women, especially African Americans and Latinxs, is an essential strategy for decreasing the rate of new HIV infections in the US (5). However, efforts in the U.S. to increase the uptake of PrEP have primarily targeted men who have sex with men (MSM), and uptake and knowledge by at-risk women remains low (5).

Effectively increasing the use of PrEP involves identifying women who would benefit from PrEP, counseling them about their risk, screening for HIV infection, lab surveillance, and offering PrEP services (6). Each step introduces different barriers to adaption into clinical care and uptake by women who are likely to benefit from PrEP. First, increasing the public's awareness of PrEP is critical. That burden is shared by governmental agencies as well as health care clinicians (7). In New York City, the Health Department implemented a 4-point plan for PrEP implementation that included PrEP promotion though social marketing campaigns targeting users and through courses and training programs targeting clinicians (8). Even with the successes achieved in the program, vigilant surveillance and monitoring was necessary when aiming for equitable impact (8).

Despite some local efforts to promote PrEP implementation in diverse practice settings, most clinicians have not provided PrEP (7, 9). Clinicians identified need for education around PrEP provision and some raise concern that addition of counseling and provision of PrEP to already busy clinical environments is challenging (10, 11). In a recent study evaluating PrEP prescription rates among women with new diagnoses of gonorrhea and syphilis, none of the female patients were prescribed PrEP (12). The authors acknowledge gaps in our understanding of the reasons for these missed opportunities and suggest that additional research is needed to understand why providers do not provide PrEP (13).

Women identified their low perceived HIV risk, negative beliefs about PrEP, and limited access to clinicians who will prescribe PrEP as the major barriers to PrEP uptake (6, 13). However, some women are open to receiving services in certain clinical environments. For example, women who received PrEP counseling in a Family Planning (F.P.) clinic were more open to taking PrEP and wanted to receive PrEP services in the F.P. clinic instead of at another health care setting (14). Finally, even after PrEP is implemented, PrEP users have reported costs and insurance as barrier to sustained use. Specifically, need for prior authorization and its associated administrative hurdles, out of pocket costs due to copayments and high deductibles and high variability in coverage by different insurance plans are challenging for some people to navigate (15).

This study aimed to identify attitudes of women of color toward PrEP and to understand their receptivity to PrEP education and initiation from their OBGYN. Understanding women's preferences can inform clinician training and service delivery necessary for improving uptake of PrEP by women at risk for HIV acquisition.

## Materials and Methods

As part of a study aimed at identifying barriers to PREP uptake among women of color, we recruited participants from an ambulatory OBGYN clinic at Montefiore Medical Center, an academic medical center in the Bronx, NY. Eligible participants were between 18 and 45 years of age, Black, Latinx or mixed race, comfortable speaking English, and able to provide informed consent.

We approached women for participation in the study at the end of their OBGYN visit from June 2018 to July 2019. We decided a priori to enroll 30 participants and purposefully selected 10 participants seeking care for family planning, 10 seeking prenatal care, and 10 seeking care for a sexually transmitted infection screening or treatment. We screened participants for HIV acquisition risk using a PrEP screening tool (see Supplementary Material). Two research team members who were not involved in the care of the patient and experienced in qualitative interview techniques conducted semi-structured interviews with all 30 participants. The interview guide included open-ended questions about recent healthcare visits, whether their clinician had counseled them about HIV prevention, their perceived HIV risk, their awareness and knowledge about PrEP, their receptivity to hearing about PrEP in an OBGYN setting, and their concerns about PrEP.

All interviews were audio-recorded, de-identified, transcribed verbatim, and then entered into Dedoose, a software program that provides a platform for managing text retrieval and analysis of qualitative data. Research team members (KA, TG-A, ARE, SH, JEM, LJB, SMD) read the first interview and identified themes and recurring ideas. After reading additional interviews, each team member identified new themes and the team drafted a provisional codebook. The team revised the codebook iteratively until the codebook reflected key themes and categories. After finalizing the codebook, each transcript was coded individually in Dedoose by research team members (KA, TG-A, ARE, SH, JEM, LJB, SMD). Coders met together, completed line-by-line coding review, and resolved coding discrepancies through consensus.

Thematic analyses used constant comparison and grounded theory approaches. We constructed matrices to compare participants' responses based on race/ethnicity, age, and degree of risk for HIV acquisition. For this specific analysis, four team members (AD, ECM, KA, SMD) undertook analyses that involved themes that informed the participants' experience with their OBGYN. In the results presented here, we identify quotes by age, race, and the type of OBGYN service they were seeking.

## Results

### Demographics

The mean age of the participants was 29.7 years (±5.4) (Table 1). The majority (60%) identified as Latinx, 33% were African American, and 7% identified as other. Seventy percent of the participants had at least one risk factor for HIV acquisition on the PrEP screening tool (see Appendix A). Only 13% had heard about PrEP before the interview. No participant had ever been prescribed PrEP.

**Table 1:** Baseline characteristics of the participants interviewed in the study.

**Characteristics**	***N* (%) or Mean (S.D.) (Range)**
Age (years)	29.7 (5.4) (20–41)
**Race/ethnicity**	
Latinx	18 (60%)
Black/African American	10 (33%)
Other	2 (7%)
**Sexual orientation**	
Heterosexual	25 (83%)
Bisexual	5 (27%)
**Relationship status**	
Married	8 (27%)
Single, never married	20 (67%)
Domestic partnership	2 (7%)
**Education**	
< High school	4 (13%)
High school/GED/Vocational training	6 (20%)
Some college	13 (43%)
Associate degree or higher	7 (23%)
Number of sexual partners in the last year	1.8 (1.4) (1–6)
1–2 partners	23 (77%)
3 or more partners	7 (23%)
**Sexually transmitted infection (STI) in the last year**	
No	24 (80%)
Yes	6 (20%)
**Number of live births among women who have ever been pregnant (*****n*** **=** **29)**	
0 live births	8 (28%)
>1 live birth	21 (70%)
**Contraception among those who do not want to get pregnant (*****n*** **=** **15)**	
Condoms only	2 (13%)
Other method (IUD, birth control pills, Depo-Provera, nexplanon, nova ring)	12 (80%)
No method	1 (7%)
**HIV testing history**	
Ever tested	29 (97%)
Never tested	1 (3%)
**Reported one or more risk factors for HIV on the PrEP screening tool**	
Yes	21 (70%)
No	9 (30%)
**Learned about PrEP from a medical professional**	
Yes	4 (13%)
No	25 (83%)
Not sure	1 (33%)
**Ever been prescribed PrEP**	
Yes	0 (0%)
No	30 (100%)

*Reasons participants screened positive: In the past 12 months had unprotected anal sex (10), diagnosed with an STI (8), partner diagnosed with an STI (4), unprotected intercourse with partner with unknown HIV status (3), unprotected intercourse with someone recently incarcerated (4).

### Thematic Analysis

**Figure 1:**
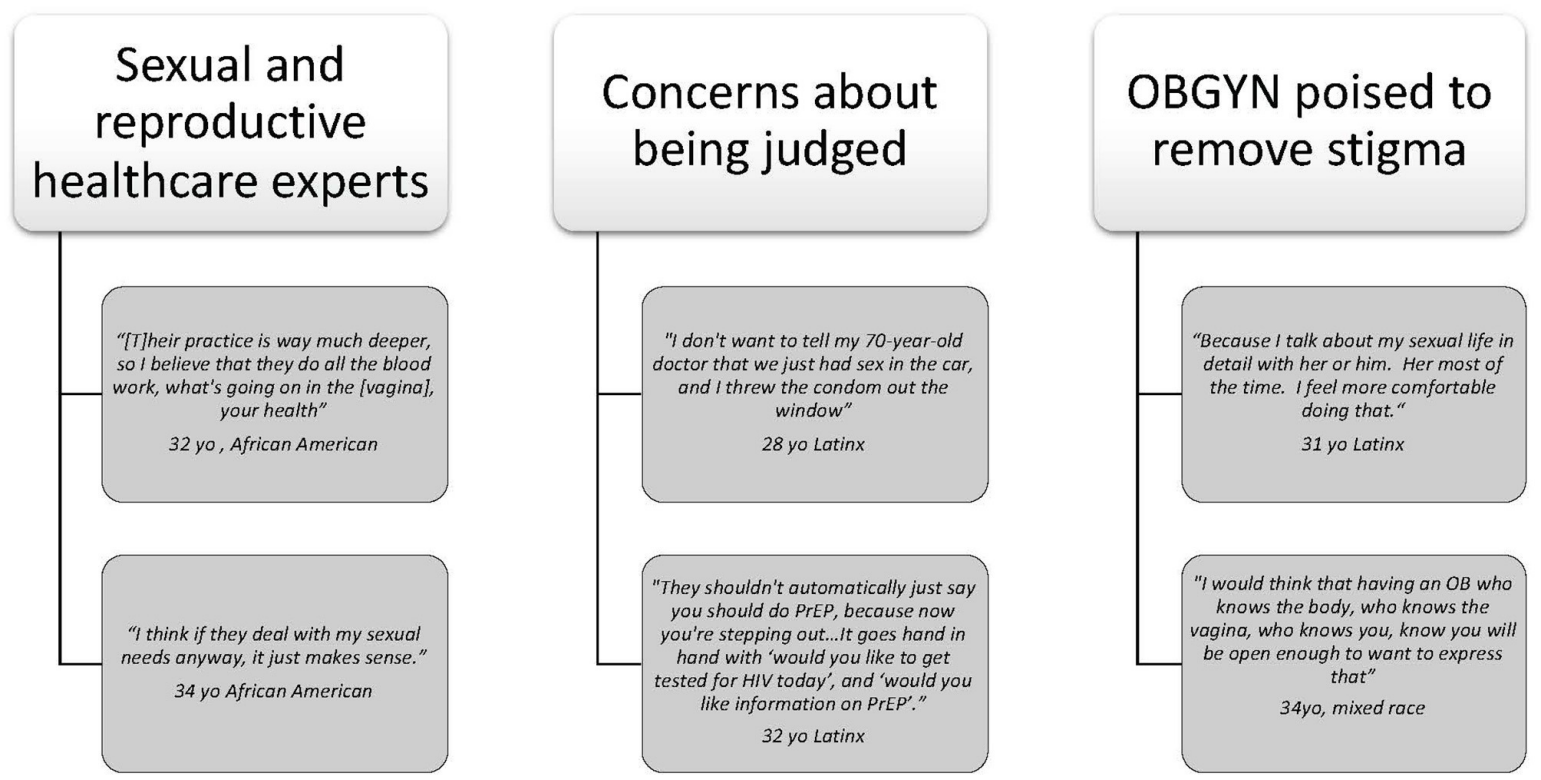
Selected quotes from three main themes.

When asked where they would prefer to receive PrEP-related services, participants identified their OBGYN as their first preference. We report here on our analysis on women's comfort discussing HIV screening, risks for HIV transmission, and provision of PrEP. Three main themes emerged: (1) participants viewed OBGYNs as experts in sexual and reproductive healthcare and therefore believed they were experts in PrEP; (2) participants were concerned about being judged by their primary care clincians if they expressed a need for PrEP; and (3) participants' trust specifically in their OBGYN enabled them to consider PrEP, even when stigma around PrEP was viewed as a barrier (Figure 1).

#### Obstetricians and Gynecologists as Experts in the Provision of Sexual and Reproductive Healthcare

Most participants named their OBGYN as the clinician they prefer to provide services related to PrEP. Participants cited trust in their OBGYN's specific knowledge and experience in women's sexual and reproductive health as a reason for this preference. One participant described this type of discussion as part of the expertise of OBGYNs:

“I feel like the OBGYN – they go, their practice is way much deeper, so I believe that they do all the blood work, what's going on in the [vagina], your health. They do more details and dig more into you, your history, your health history, so I feel like they should most of all be the one …to tell their patients.” (32-year-old, African American, prenatal care clinic)

Other participants felt that PrEP counseling and provision falls naturally within the scope and skill set of the OBGYN, particularly the screening, counseling, and treatment of sexually transmitted infections (STIs) they already receive from their OBGYN.

“They're dealing with the areas specifically that would be harmed from getting [HIV]. You know what I mean… I think if they deal with my sexual needs anyway, it just makes sense.” (34-year-old, African American, Family Planning clinic)

Several women specifically mentioned prenatal care as an essential window for sexual health counseling with their OBGYN. One participant described her experience with prenatal care as a time of candidness with her clinician, despite having had previous stigmatizing healthcare experiences related to a diagnosis of genital herpes.

“… because of my situation being pregnant right now I'm very open with them [my OBGYN]. Because I want to know everything. Yes, I need to know everything.” (27 -year-old, Latinx, STI clinic)

Another participant had a different experience and noted that HIV-related counseling was noticeably absent during preconception counseling and prenatal care.

[A doctor talked about HIV prevention] “every time I go, except when I got pregnant. That's when they just stopped.” (22-year-old, Latinx, STI clinic)

#### Concern About Being Judged by Clinicians If They Expressed Interest in PrEP

When discussing potential screening and counseling opportunities for PrEP with various healthcare clinicians, participants voiced concerns about stigma. Several participants worried that they might be judged about their sexual behaviors if they inquired about PrEP.

“I think they would be thinking, 'What is she doing?' Cause they are old school… they be thinking you running wild and having all these partners” (32-year-old, African-American, prenatal care clinic)

“I don't want to tell my 70-year-old doctor that 'We just had sex in the car, and I threw the condom out the window…' Their face is going to be like, 'Um, get out of my office.”' (28-year-old, Latinx, family planning clinic)

Apprehension about feeling judged led some women to withhold information needed to identify risk for HIV infection. One participant described how perceptions of feeling judged limited her willingness to talk about PrEP. She was preoccupied with observing her clinician closely for signs of judgment.

“Unless they [are] judgmental. That's when I'm concerned. Like I step back. [Judgmental in] their face reaction…like raising an eyebrow, open their eyes big…they're not trying to show it, but I could see it.” (22-year-old, Latinx, STI clinic)

Women mentioned strategies they used to avoid possible stigma from the physician. One participant explained that she would prefer to go to a clinic where she is not known:

[I would want to get PrEP from] “my GYN clinician or those remote locations where nobody knows me..., and I was going there [because] they begin labeling right away. These remote locations that can just check-in, go in for it, or get a refill without anyone knowing.” (29-year-old, Latinx, Family Planning clinic)

For this participant, if she could not get care from her trusted OBGYN, she would seek care in a more anonymous setting to avoid feeling shamed and stigmatized. Other participants supported this idea of universal counseling. Universal screening and STI testing would destigmatize PrEP by placing the care in the context of preventive health.

“I think that that's mostly just like not talking about HIV so much but just the benefits of the prevention of HIV and just overall. Like the same way you have to get vaccines to go to a public school… just see it as a bigger deal than it is. It's preventative.” (28-year-old, Latinx, family planning clinic)

“They shouldn't automatically just say you should do PrEP, because now you're stepping out…It goes hand in hand with ‘would you like to get tested for HIV today', and ‘would you like information on PrEP'. It should be something that it's [available]…but it's not shoved in your face because you stepped out, so you should do this.” (32 years old, Latinx, STI clinic)

OBGYN clinicians can reduce potential stigmatizing experiences by employing a universal approach to PrEP counseling and giving patients a choice to opt in or out of receiving the information.

“Everybody should be familiarized or at least know about it… and they have the choice whether to hear more about it or not. I think it should be offered to everybody and let them decide if they want to hear more about it or if not.” (29-year-old, Latinx, prenatal care clinic)

In addition to their concern about potential stigma from their OBGYN, several women said that being on PrEP itself is stigmatizing as it confers a label of sexual promiscuity. Participants suggested that doctors could help mitigate PrEP stigma by addressing it directly:

“[Let] them know that [PrEP] is a way that they can have control over their health, that it doesn't mean that they're promiscuous. Because I always feel that's the perception people are going to have.” (29-year-old, Latinx, prenatal care)

“The first thing I would mention is taking PrEP doesn't make you a whore, doesn't make you a slut, doesn't make you a bad person… because that's what people automatically assume.” (27-year-old, Latinx, STI clinic)

#### Trust in Their OBGYN Helps Overcome Stigma as a Barrier

Participants wanted to have open and honest conversations with their OBGYN but worried about feeling “singled out” if their disclosures resulted in the recommendation of PrEP. If the conversation became normalized as part of every routine reproductive healthcare visit, the recommendations seemed less biased to the patient.

[If] “it's a regular routine visit, it's a little bit easier to discuss something, because it's more a 'let's see what we find today.' As opposed to a 'I don't feel right and I'm checking for something', then it's more of a they're already in their head trying to figure out what you have. I feel they're already judging you.” (27-year-old, Latinx, STI clinic)

Despite their worry about being stigmatized by clinicians during conversations about PrEP, participants consistently emphasized the importance of being counseled about PrEP and most were comfortable discussing their sexual health history and concerns with their OBGYN clinician.

“Because I talk about my sexual life in detail with her or him. Her most of the time. I feel more comfortable doing that. I'm on birth control, I am on top of my testing, I am on top of my visits. I feel like it would be appropriate for the GYN to administer PrEP services.” (31-year-old, Latinx, Family Planning clinic)

Many participants stated that they would only be comfortable hearing about PrEP from their clinician if he or she was non-judgmental. OBGYNs emerged as trusted clinicians who combine in-depth knowledge of women's health with non-judgmental compassion that allowed women to overcome potential stigma related to discussions of PrEP.

“I would think that having an OB who knows the body, who knows the vagina, who knows you, know you will be open enough to want to express that so that's what she's there for in my opinion.” (34 years old, mixed race, Prenatal Care clinic)

## Discussion

In this study of women attending OBGYN clinics at an urban medical center, we found that women's comfort discussing PrEP with their OBGYN were intertwined with the trust in their clinician as well as the stigma they confront about their sexuality, particularly sexual behavior that puts them at risk for HIV infection. Their OBGYN may be the only person they can be completely honest with about their sexual behavior. Yet, there was the lingering worry that even their OBGYN, the safe harbor for their sexual concerns, may judge them. Their preoccupation with how their clinician perceived them represents a possible barrier to the provision of PrEP. Many women said that OBGYNs are experts in sexual and reproductive healthcare and should offer PrEP services because prevention of HIV is a natural extension of care they are already receiving or expect to receive. While pregnancy is still an opportunity to offer PrEP, OBGYNs have not advanced HIV prevention with PrEP in this clinical setting.

Our study participants consistently agreed that HIV prevention falls within the scope of sexual and reproductive health care addressed and managed by an OBGYN. However, as we discussed PrEP and who should consider it, women used language that indicated “self-stigma”, which is a barrier to identifying those who might benefit from PrEP. Self-stigma is a phenomenon where an individual internalizes feelings of shame associated with a socially devalued identity (16) and has been documented as one of the biggest challenges to HIV prevention (17). We saw examples of this when they described being hypervigilant about how a clinician reacted to their sexual behavior or selected them for a PrEP conversation. Few saw the assessment of risk behavior as a neutral interaction with their clinician; most associated it with an underlying judgment of sexual promiscuity.

Women's experience of being judged for their sexual behaviors is grounded in the sexual double standard. Heterosexual men gain a positive reputation for being sexually active and dominant. In contrast, women risk hurting their social standing through sexual activity (18, 19). The sexual double standard seems to be part of the shame felt by study participants. Sexual double standard constitutes a significant barrier to PrEP access because it fuels self-stigma. If women disclose sexual behaviors that put them at risk for HIV to gain access to PrEP, they risk feeling ashamed and judged. Women in relationships that risk exposure to sexually transmitted infections who choose to remain in those relationships are also subjecting themselves to the clinician's judgment. When they do not identify their sexual behaviors and their risk of acquiring HIV, they do not have the opportunity for prevention afforded by PrEP.

Even women in the study believed that their freedom in sexual behavior is normal and healthy, fear what others will think, including their OBGYN. This anticipated stigma (20) prevents them from revealing their sexual history or their concerns about the risks posed by their partners and could prevent them from availing themselves of the opportunity to take PrEP. However, when they perceived their OBGYN to be open and non-judgmental, they were less fearful of honestly discussing their sexual histories. Women told us they needed a “judgment-free zone” to talk about PrEP. They believed that OBGYNs are the most knowledgeable and least judgmental clinicians they see and thus best suited to be their PrEP clinician. Women in our study were confident in OBGYNs' knowledge and expertise, believed them to be trustworthy and perceived them as the best clinician to prescribe PrEP. These findings are like those reported by Auerbach and colleagues, who found that women preferred hearing about PrEP from their OBGYN but acknowledged concern about possible stigma (21).

Research with MSM has identified similar concerns to those described by our study participants regarding stigma as a barrier to PrEP uptake (22–25). MSM share the same concern about facing stigma around PrEP from their clinician, limiting their willingness to engage in conversations about their sexual health and risks of HIV acquisition (24, 25). Even when some MSM expressed a desire to use PrEP, they have felt shamed or dismissed by the clinician (25). The importance of how sexual behaviors are perceived, and the impact on PrEP use, have led to the promotion of a sex-positive approach to increasing PrEP use among at-risk groups, specifically targeting African American and Latinx MSM. Sex-positivity as a means of affirming pleasure and desire in gender and sexual minority communities (26) could also be adopted as an approach for increasing PrEP use among women.

For MSM, but less for women, there have been significant public health efforts to increase PrEP uptake. In order to address the issue of stigma among MSM, researchers examined the role of language around PrEP and how changing the language could decrease stigma around PrEP use and increase uptake (24). Golub suggested changing the language to describe PrEP as a benefit for everyone which is consistent with the updated CDC guidelines for HIV prevention (24, 27). Modifying screening tools to assess a person's sexual health concerns and goals and prioritize sexual health over concern about risk and risk compensation would be helpful (24). This approach could also be appropriate for women. Women in this study recommended that PrEP screening and discussion should be universal and part of general sexual and reproductive health care for all OBGYN patients. Targeting particular women for a PrEP conversation based on a screen introduces potential stigma and bias and negatively influences women's willingness to discuss PrEP. Golub's recommendation includes adapting the language used by the American College of Obstetricians and Gynecologists (ACOG) to frame the importance of contraception (24). An adapted statement might read: A sexual health plan is a set of personal goals regarding how to engage in fulfilling sexual expression while preventing HIV infection and other sexually transmitted infections based on individual priorities, resources, and values (13).

By taking a universal approach to screening, patients would have more autonomy. They could avoid feeling judged in discussions about PrEP than if their specific sexual histories were the catalyst for the discussion. Calabrese presented a care model that is in line with our participants' expressed desires for PrEP integration into OBGYN care that could improve PrEP access and equity (28). In her model, all adolescents and adults are offered PrEP unless medically contraindicated and without consideration of demographic and behavioral characteristics (28). Clinicians can offer additional counseling if specific risk factors are present but that maintaining a universal approach allows more patient decision making (28). But for some OBGYNs, the ability to add PrEP provision to routine care may seem challenging for OBGYNs that already face challenges with providing multiple components of care with significant time constraints (10). Frameworks such as RE_AIM (Reach, Efficiency, Adoption, Implementation, Maintenance) have been used to successfully integrate HIV Prevention services despite known barriers like time constraints and insurance barriers (9).

This study adds to the growing body of literature that has identified a gap in HIV prevention for women who might benefit from PrEP and supports universal screening and counseling for PrEP as part of reproductive health care. A strength of the study includes the inclusion of underrepresented women and women at the highest risk of HIV acquisition.

There are some limitations to this study. Women were recruited in the OBGYN clinics and therefore were already engaged in care with an OBGYN. Their relationship with those OBGYNs may reflect how they see PrEP provision as an extension of the care they are already receiving. It is unclear whether women who do not have an OBGYN would agree that an OBGYN should prescribe PrEP. Still, our findings are important for many women, specifically younger reproductive-aged women who engage in OBGYN care, which may be their only contact with the healthcare system and would represent their first or only opportunity to learn about PrEP.

PrEP is an important addition to comprehensive women's health care. In their discussion of counseling on PrEP, participants offered insight into approaches that could reduce potential experiences of stigma. Multiple participants suggested that initial counseling on PrEP should be offered universally to patients, regardless of sexual history or personal risk factors. In this way, the conversation is framed as a general effort to increase patient awareness and access to information rather than “targeted” counseling to women who are “judged” to be at risk. Women's concerns about feeling judged, as exemplified through their descriptions of clinicians making associations with the need for PrEP and promiscuity, as well as their desire to seek different clinic locations, is a significant barrier to acceptance of PrEP. However, through their descriptions of their relationship with their OBGYN clinicians, this barrier can most likely be surmounted by the OBGYN.

## Data Availability Statement

The raw data supporting the conclusions of this article will be made available by the authors, without undue reservation.

## Ethics Statement

The studies involving human participants were reviewed and approved by the Institutional Review Board at Albert Einstein College of Medicine. The patients/participants provided their written informed consent to participate in this study.

## Author Contributions

JM and LB conceived and designed the study. JM, LB, KA, and SD collected the data. KA, TG-A, AE, SH, JM, LB, and SD analyzed and coded the data. EC, AD, KA, and SD interpreted the data. AD drafted the final manuscript in collaboration with EC, SD, and KA. All authors read and approved the final manuscript.

## Funding

Funds to support this project were provided as a supplement from the Office of Women's Health to 3R01MH107297-03S1, PC4PrEP: Integrating PrEP into Primary Care. SH and JM were also supported by a NIMH Center Grant P30-MH43520 (Principal Investigator: Robert H. Remien, PhD).

## Conflict of Interest

The authors declare that the research was conducted in the absence of any commercial or financial relationships that could be construed as a potential conflict of interest.

## Publisher's Note

All claims expressed in this article are solely those of the authors and do not necessarily represent those of their affiliated organizations, or those of the publisher, the editors and the reviewers. Any product that may be evaluated in this article, or claim that may be made by its manufacturer, is not guaranteed or endorsed by the publisher.
