# Supplementary material for: Women Trust Their OBGYNs to Provide Preexposure Prophylaxis: An Opportunity for HIV Prevention
Source: Front Reprod Health. 2022 Mar 15;4:832287. doi: 10.3389/frph.2022.832287 (PMC9580770; doi:10.3389/frph.2022.832287)
Supplement: Supplementary file 1 [file Table_1.DOCX]

**Women and PrEP Pre-screening Tool**

| **Please check the box that best represents your answer (ONE answer per question).** | **NO** | **YES** | **Don’t Know** |
| --- | --- | --- | --- |
| **In the past 12 months,** have you had vaginal sex (putting the penis into the vagina) without a condom? |  |  |  |
| **In the past 12 months**, have you had anal sex (putting the penis or having the penis put into the butt) without a condom? |  |  |  |

| **Over the past 12 months**, think about all the persons you have had sex with without using a condom. Were any of them someone who……… | **NO** | **YES** | **Don’t Know** |
| --- | --- | --- | --- |
| is HIV-positive |  |  |  |
| you don’t know their HIV status |  |  |  |
| had a sexually transmitted infection (STI) |  |  |  |
| might have had HIV or a STI |  |  |  |
| gave or received money, food, or shelter to have sex |  |  |  |
| injected drugs or shared needles |  |  |  |
| was in jail or prison in the past 12 months |  |  |  |
| was a man who has sex with other men |  |  |  |

| **In the past 12 months**, have you ……… | **NO** | **YES** | **Don’t Know** |
| --- | --- | --- | --- |
| been told by a doctor or nurse that you had a STI such as chlamydia, gonorrhea, syphilis, herpes, or HPV/genital warts? |  |  |  |
| been given PEP (Post-exposure Prophylaxis)? PEP is HIV drugs that are prescribed by a provider “AFTER” being potentially exposed to HIV to prevent becoming infected. |  |  |  |
| have you injected drugs? |  |  |  |

|  | **NO** | **YES** | **Don’t Know** |
| --- | --- | --- | --- |
| Are you worried or concerned about your risk for getting HIV or a STI? |  |  |  |
